# Ultrasound Particle Image Velocimetry to Investigate Potential Hemodynamic Causes of Limb Thrombosis After Endovascular Aneurysm Repair With the Anaconda Device

**DOI:** 10.1177/15266028231219988

**Published:** 2023-12-27

**Authors:** Hadi Mirgolbabaee, Lennart van de Velde, Robert H. Geelkerken, Michel Versluis, Erik Groot Jebbink, Michel M. P. J. Reijnen

**Affiliations:** 1 Multi-Modality Medical Imaging Group, Technical Medical Centre, University of Twente, Enschede, The Netherlands; 2 Physics of Fluids Group, Technical Medical Centre, University of Twente, Enschede, The Netherlands; 3 Department of Vascular Surgery, Rijnstate Hospital, Arnhem, The Netherlands; 4 Section of Vascular Surgery, Department of Surgery, Medisch Spectrum Twente, Enschede, The Netherlands

**Keywords:** limb thrombosis, endovascular aneurysm repair, in vitro hemodynamic quantification, high–frame-rate imaging, contrast-enhanced ultrasound, particle image velocimetry, vector complexity, residence time

## Abstract

**Purpose:**

To identify potential hemodynamic predictors for limb thrombosis (LT) following endovascular aneurysm repair with the Anaconda endograft in a patient-specific phantom.

**Materials and Methods:**

A thin-walled flow phantom, based on a patient’s aortic anatomy and treated with an Anaconda endograft, that presented with a left-sided LT was fabricated. Contrast-enhanced ultrasound particle image velocimetry was performed to quantify time-resolved velocity fields. Measurements were performed in the same phantom with and without the Anaconda endograft, to investigate the impact of the endograft on the local flow fields. Hemodynamic parameters, namely vector complexity (VC) and residence time (RT), were calculated for both iliac arteries.

**Results:**

In both limbs, the vector fields were mostly unidirectional during the peak systolic and end-systolic velocity phases before and after endograft placement. Local vortical structures and complex flow fields were observed at the diastolic and transitional flow phases. The average VC was higher (0.11) in the phantom with endograft, compared to the phantom without endograft (0.05). Notably, in both left and right iliac arteries, the anterior wall regions corresponded to a 2- and 4-fold increase in VC in the phantom with endograft, respectively. RT simulations showed values of 1.3 to 6 seconds in the phantom without endograft. A higher RT (up to 25 seconds) was observed in the phantom with endograft, in which the left iliac artery, with LT in follow-up, showed 2 fluid stasis regions.

**Conclusion:**

This in vitro study shows that unfavorable hemodynamics were present mostly in the limb that thrombosed during follow-up, with the highest VC and longest RT. These parameters might be valuable in predicting the occurrence of LT in the future.

**Clinical Impact:**

This in-vitro study aimed to identify potential hemodynamic predictors for limb thrombosis following EVAR using ultrasound particle image velocimetry (echoPIV) technique. It was shown that unfavorable hemodynamic norms were present mostly in the thrombosed limb. Owing to the in-vivo feasibility of the echoPIV, future efforts should focus on the evaluation of these hemodynamic norms in clinical trials. Thereafter, using echoPIV as a bedside technique in hospitals becomes more promising. Performing echoPIV in pre-op phase may provide valuable insights for surgeons to enhance treatment planning. EchoPIV is also applicable for follow-up sessions to evaluate treatment progress and avoid/predict complications.

## Introduction

Limb thrombosis (LT) is a common complication following endovascular aneurysm repair (EVAR).^1,2^ In a meta-regression analysis of 5454 patients, Hammond et al^3^ demonstrated a prevalence of 5.6%. Patients suffering from LT may be asymptomatic but do often develop symptoms such as intermittent claudication or acute limb–threatening ischemia, impacting the quality of life and therefore LT regularly requires a reintervention.^4–6^ Cochennec et al^4^ reported that 83.3% of the LTs were symptomatic, and only 11.1% were asymptomatic.

The Anaconda limb (Terumo Aortic, Inchinnan, Scotland) is designed with multiple independent rings, to enhance its flexibility and to prevent kinking; however, it has been related to a high LT rate between 3.5% and 9.8%.^7–11^ Rödel et al^9^ reported a decrease in the LT rate from 13.1% to 6.4%, when comparing the second and third generations, respectively. Midy et al^8^ reported a LT rate of 7.9% using this third generation. Nonetheless, the underlying reasons behind Anaconda’s high LT rate are not yet fully understood.

Potential causes and predictors of LT after EVAR can be categorized into endograft-related and anatomical (ie, patient-related) parameters. Kinking and fabric infolding were identified as important endograft-related predictors for LT.^12,13^ Moreover, a small iliac artery diameter,^14^ iliac artery tortuosity,^6,12,13^ the presence of calcifications,^5,12,13^ and iliac artery angulation^5,12^ are anatomical factors that may induce LT. However, the relationship between these anatomical and endograft-related factors, eventually causing LT, is not yet fully understood.

Previously, Simmering et al^15^ elaborated on a concertina effect hypothesis as a potential risk factor for LT, stating that neighboring stent ring distances decrease in tortuous and/or shortened Anaconda limbs, which may cause inward folding of the graft fabric between them. The independent rings, with their cribs and valleys design, may lead to local endograft invagination in tortuous anatomy. Moreover, with sac shrinkage not only the diameter but also the length of the abdominal aortic aneurysm (AAA) might decrease, which could result in kinking or compression of the limbs. These situations, in turn, may induce unfavorable local flow patterns that could lead to stasis of blood, thrombus formation, and eventually LT.^15^

We studied local hemodynamics by quantifying vector complexity (VC) and residence time (RT) based on the obtained velocity field in a patient-specific phantom. Vector complexity defines the unfavorable flow pattern in the investigated region of lumen, by distinguishing between unidirectional and fully complex flow.^16^ Residence time simulation utilizes a Lagrangian approach to assess thrombus formation probability by determining adverse flow regions in the lumen.^17,18^ Hence, VC and RT were calculated in iliac arteries of a phantom that was derived from a patient that presented with a left-sided LT 2 years after EVAR using the Anaconda endograft to evaluate their potential relation with that patient’s LT event.

## Materials and Methods

### In Vitro Model

**Figure 1.**
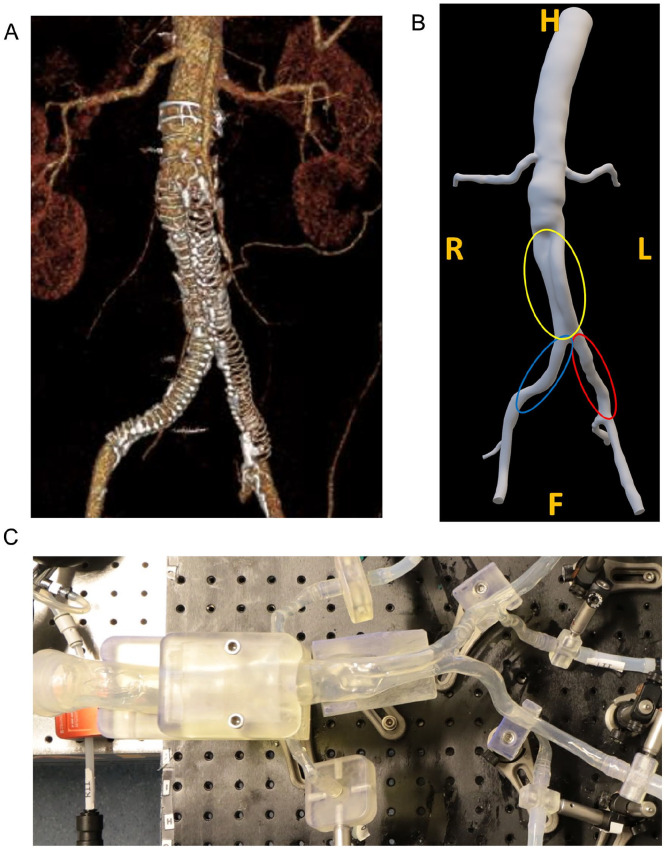
(A) Anaconda endograft in situ configuration after limb thrombosis in left limb, and (B) segmented anatomy, based on the last follow-up computed tomography angiography before limb occlusion in the left limb. Left and right common iliac arteries are marked by red and blue colors, respectively. The fused neo-iliacs lumina are marked by yellow, mimicking the pre-op abdominal aorta’s flow lumen inside abdominal aortic aneurysm sac. Directions are marked in orange. (C) Fabricated thin-walled patient-specific phantom. H, head; F, foot; R, right; L, left.

A phantom was designed based on a patient’s aortoiliac anatomy (Figure 1A), who developed a left-sided LT 26 months after EVAR with an Anaconda endograft (see Supplemental Information [SI] 1 for anatomy and endograft details). The last computed tomography angiography (CTA) scan before LT, made 21 months after EVAR, was used to perform flow lumen segmentation and create a 3D model (Figure 1B) using SimVascular software (Version 2021.02, Stanford University, Stanford, California). Thereafter, a silicone-based thin-walled (~1.5–2.0 mm wall thickness) flexible phantom (Figure 1C) was produced (Elastrat Sarl, Geneva, Switzerland). There were no significant changes comparing the pre-op and post-op anatomies of the iliac arteries (see SI 1). Also, as a necessity for the phantom fabrication process, the post-op neo-iliacs lumina had to be fused, which resembled the pre-op abdominal aorta’s flow lumen inside AAA sac (marked by yellow color in Figure 1B). Therefore, it was decided to initially perform ultrasound particle image velocimetry (echoPIV) measurements in the unstented phantom (referred to as before endograft placement [BE]) to represent pre-op condition. Thereafter, since the primary goal was to quantify unfavorable hemodynamic conditions prior to LT, an Anaconda endograft was placed by an experienced vascular surgeon (R.H.G.), with equal sizing as was used in the patient, and echoPIV measurements were repeated in the stented phantom (referred to as after endograft placement [AE], see SI 1 for endograft details).

### In Vitro Setup

**Figure 2.**
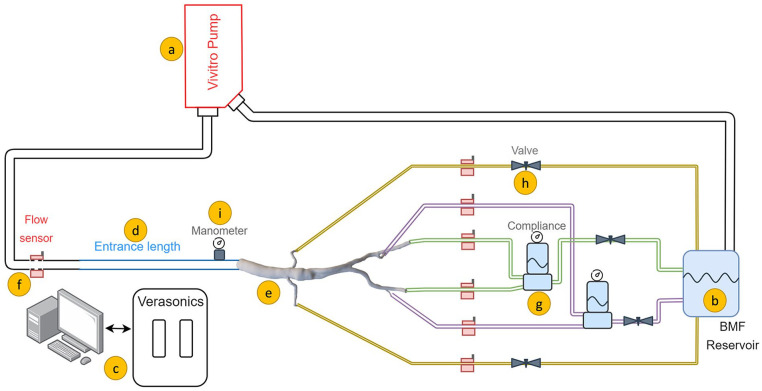
Schematic of the in vitro flow setup, consisting of (A) pulsatile pump, (B) blood mimicking fluid (BMF) reservoir in which the microbubbles were injected, (C) Vantage 256 Verasonics system, (D) inflow rigid pipe ensuring fully developed Womersley flow, (E) patient-specific phantom, (F) ultrasonic flow sensors, (G) compliance to generate backward flow, (H) adjustable valve to create resistance, and (I) analogue manometer.

The phantom was placed in a flow system, based on a second-order Windkessel design (Figure 2). A blood mimicking fluid (BMF) was used,^19^ consisting of a mixture of 45% w/w glycerol (Fisher Scientific, Landsmeer) and 55% w/w phosphate-buffered saline solution (Sigma-Aldrich, Merck Life Science, Amsterdam). The average density and kinematic viscosity of the BMF solution were 1115.25±0.25 kg/m^3^ and 3.95±0.03 mm^2^/s, respectively, measured using a density meter (Anton Paar GmbH, Graz) and Ubbelohde viscometer (Xylem Analytics, Weilheim). A hydraulic piston-driven pump (SuperPump, ViVitro labs, Victoria) was used to set a suprarenal flow profile as inlet boundary condition. The suprarenal flow waveform was constructed by modifying the average supraceliac flow profile of patients with an AAA.^20^ The supraceliac profile was converted to a suprarenal profile under assumption that 65% of the supraceliac flow go to the suprarenal branches.^21^

A 120 cm inlet pipe was used to guarantee a fully developed Womersley inlet flow profile.^22^ Renal artery flows were regulated using resistances. Meanwhile, the outflow via the internal/external iliac arteries was coupled to the flow circuit using a compliance and resistance to reproduce typical iliac triphasic flow patterns. Bi-directional clamp-on ultrasound flow sensors (CO.55; Sonotec, Halle) were mounted to monitor flow profiles in inflow and outflows. Pressure was monitored using analogue manometers (Welch Allyn, Inc, Skaneateles Falls, New York). The flow circuit was kept pressurized at 120/80 mm Hg using the outflow resistances. Furthermore, the study was carried out under human resting condition at 60 beats/min pulsation while satisfying the outflow boundary conditions by matching the outflow volumetric flow rate distributions (ie, one fourth to each renal artery, and one fourth to each combined internal/external iliac arteries).^21^

### Flow Visualization

Ultrasound particle image velocimetry measurements were performed using high–frame-rate (HFR) contrast-enhanced plane wave ultrasound (CEUS) programmed with a Vantage 256 research US system (Verasonics, Kirkland, Washington). The region of interest (ROI) for the BE and AE echoPIV measurements was the right and left common iliac arteries and graft limbs distal to the aortic bifurcation, respectively (Figure 1B). Ultrasound contrast microbubbles (SonoVue; Bracco, Milan) were injected into the BMF reservoir; thereafter, HFR-CEUS images were captured using an L12-3V linear probe (Verasonics, Kirkland, Washington) at its center frequency (7.5 MHz). Singular value decomposition clutter filtering was used to preprocess the radiofrequency data prior to image reconstruction (see SI 2).^23^

### PIV and Hemodynamic Analysis

B-mode US images were masked and used as input for PIV analysis. PIVlab V2.02 toolbox (MATLAB R2019b; MathWorks Inc, Natick, Massachusetts) was used to perform a 4-pass cross-correlation technique with 50% window shifting and spline deformation.^24^ Local median filter, temporal ensemble averaging, and cardiac averaging were used to post-process the obtained PIV velocity fields.

**Figure 3.**
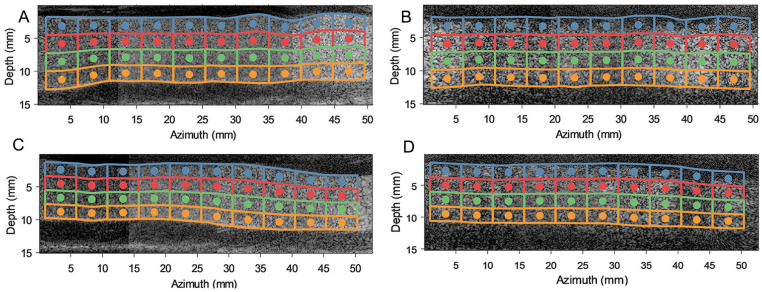
Before stenting and after stenting contrast-enhanced plane wave ultrasound images of the iliac arteries. The flow lumen is divided into 4 equally spaced vertical regions based on lumen’s centerline, with each region divided into 10 regions of interest (based on the average Anaconda ring distances). These regions are named “anterior wall” (blue windows), “anterior middle” (red windows), “posterior middle” (green windows), and “posterior wall” (orange windows). (A) Before endograft anatomy of left iliac artery. (B) After endograft anatomy of left iliac artery. (C) Before endograft anatomy of right iliac artery. (D) After endograft anatomy of right iliac artery.

Two hemodynamic parameters were computed based on the obtained velocity field, including VC, quantifying the overall flow complexity of a chosen ROI within the lumen based on the spread of the velocity field directions.^16^ Vector complexity has a value from zero to one, where zero corresponds to unidirectional flow and one refers to fully complex flow with a maximal spread in velocity vector directions. To calculate the VC, flow lumens were initially partitioned into 40 ROIs, by using their centerline and average AE ring distances in the horizontal and vertical directions, as depicted in Figure 3 (see SI 2 for more details about the VC implementation). Residence time was computed to quantify potential regions for thrombus formation by distributing mass-less seed particles, resembling red blood cells within the flow lumen, and tracking their motions based on the obtained velocity fields. Residence time was then defined as the time elapsed for all uniformly seeded mass-less particles to exit defined regions of the lumen.^17^ If these particles adhere to a specific segment of the lumen (eg, recirculation and/or stagnation zones), thereby causing an increase in the RT value, such a region can be designated as an adverse flow area, which in turn may contribute to the occurrence of thrombus formation^18^ (see SI 2 for more details about the RT implementation).

## Results

### Velocity Fields

**Figure 4.**
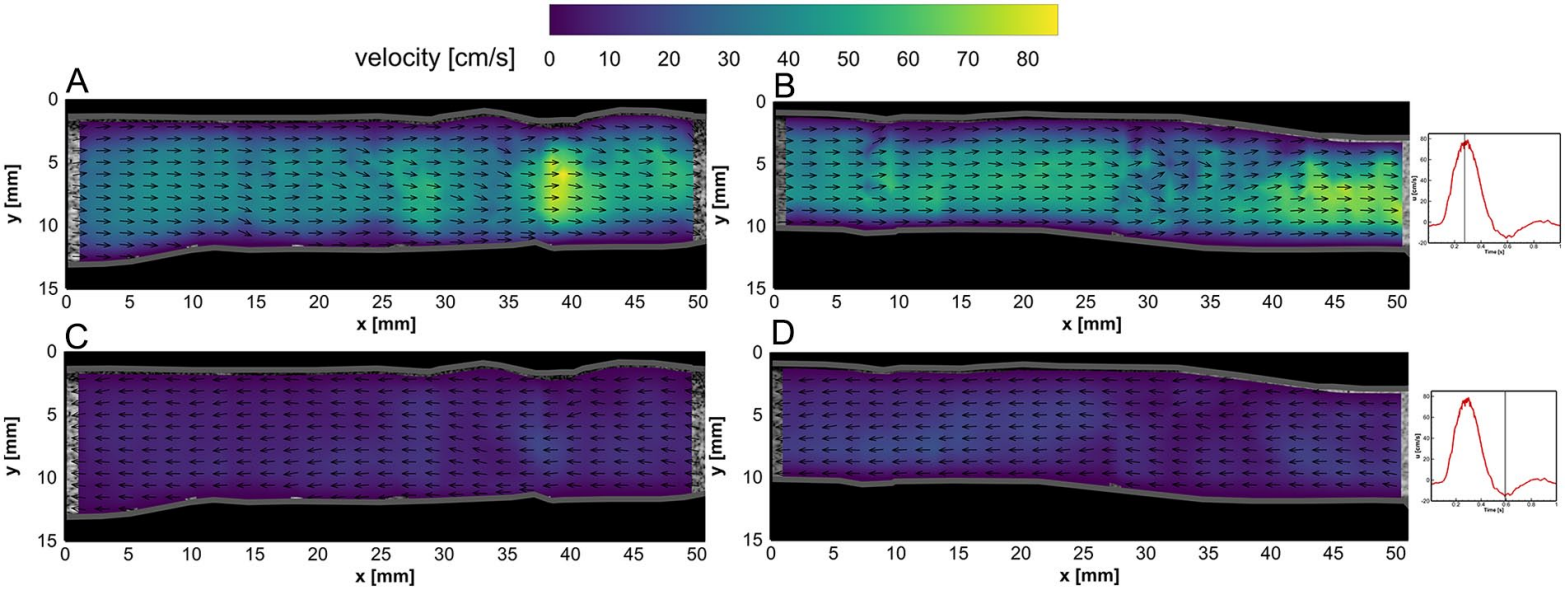
Flow fields in the left iliac artery at (A) peak systolic velocity (PSV) and (C) end-systolic velocity (ESV) time points before endograft placement. Flow fields in the right iliac artery at (B) PSV and (D) ESV time points before endograft placement. Velocity vector magnitudes are depicted by the color bar, while vector lengths are kept equal for better visualization of flow direction (refer to videos 1 and 2 in SI 3).

**Figure 5.**
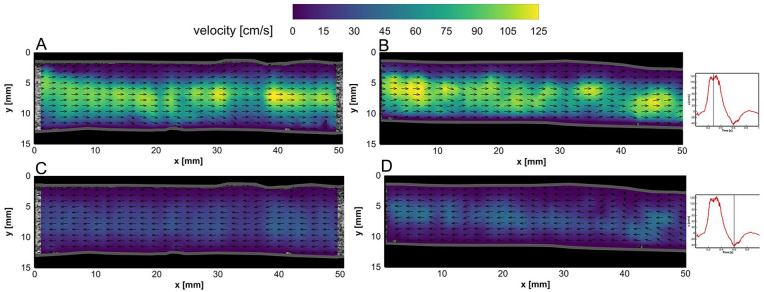
Flow fields in the left iliac artery at (A) peak systolic velocity (PSV) and (C) end-systolic velocity (ESV) time points after endograft placement. Flow fields in the right iliac artery at (B) PSV and (D) ESV time points after endograft placement. Velocity vector magnitudes are depicted by the color bar, while vector lengths are kept equal for better visualization of the flow direction (refer to videos 3 and 4 in SI 3).

The BE flow fields at peak systolic velocity (PSV) and end-systolic velocity (ESV) time points are depicted for both left and right iliac arteries in Figure 4A–D. Similarly, AE flow fields in left and right graft limbs at PSV and ESV time points are illustrated in Figure 5A–D. Two-dimensional (2D) velocity vector fields over an averaged cardiac cycle of BE and AE cases are shown in supplemental videos 1 to 4 (see SI 3).

In both BE and AE cases, the vector fields were mostly uniform during systolic (~0.10–0.45 seconds) and near the ESV (ie, maximum backward flow) phase, whereas local vortical structures and complex flow fields were observed at diastolic (~0.7–1.0 seconds) and flow reversal phases, during which the flow switched from forward to backward directions and vice versa (refer to videos 1–4 in SI 3). Higher flow velocities were obtained in the center of the lumens. The AE center luminal velocity magnitude at PSV (ie, maximum forward flow) and ESV phases was noticed to increase up to 40 and 20 cm/s in comparison with BE velocity fields, respectively.

### Vector Complexity

**Figure 6.**
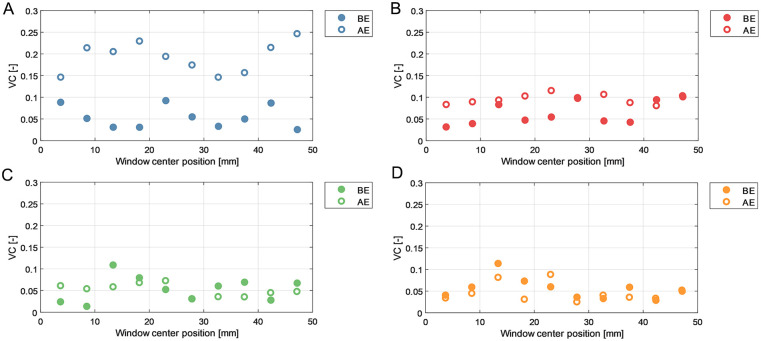
Vector complexity (VC) results over different regions of the left iliac artery before endograft placement (BE) and after endograft placement (AE). (A) Anterior wall window. (B) Anterior middle window. (C) Posterior middle window. (D) Posterior wall window.

**Figure 7.**
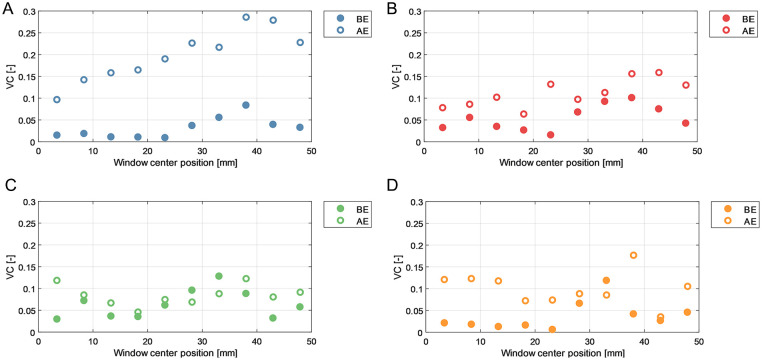
Vector complexity (VC) results over different regions of the right iliac artery before endograft placement (BE) and after endograft placement (AE). (A) Anterior wall window. (B) Anterior middle window. (C) Posterior middle window. (D) Posterior wall window.

Vector complexity results for left and right iliac arteries are depicted in Figures 6 and 7, respectively. Average VC values are summarized in Table 1 for anterior (ie, average of blue and red windows) and posterior (ie, average of green and orange windows) walls.

**Table 1.** Average VC in Iliac Arteries During BE and BE Experiments.

	Average VC	
	BE	AE
Left iliac		
Anterior wall	0.06	0.14
Posterior wall	0.05	0.05
Right iliac		
Anterior wall	0.04	0.15
Posterior wall	0.05	0.09

All values were rounded to 2 decimal points.

Abbreviations: AE, after endograft placement; BE, before endograft placement; VC, vector complexity.

In both sides the VC was higher in the anterior wall region as opposed to the posterior wall region, in both the BE and AE measurements. In addition, VC in the right iliac artery showed an increasing trend in complexity in the flow direction, implying that downstream flow is associated with less unidirectional flow. The analysis revealed a higher average VC in AE flow fields compared to BE cases. Notably, in both left and right iliac arteries, the anterior wall windows (blue windows in Figure 3) corresponded to a maximal 4- and 6-fold increase in VC values in AE flow fields, respectively.

### Residence Time

**Figure 8.**
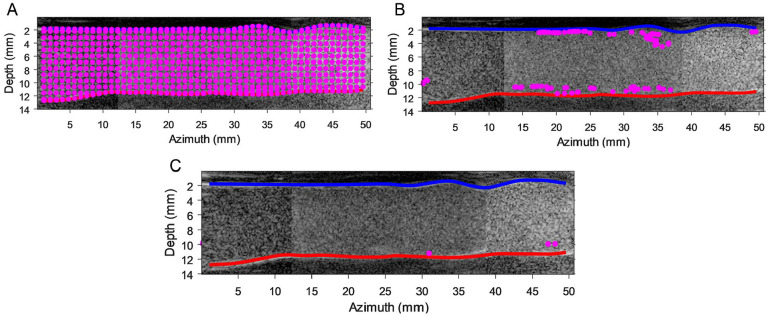
Particle trajectory simulations performed using particle image velocimetry velocity fields before endograft placement on left iliac arteries at different time points. (A) *t*=0 seconds, (B) *t*=1 seconds, and (C) *t*=5.3 seconds. The blue and red curves represent the anterior and posterior lumen wall, respectively. SI 3 contains animations of all residence time simulations (supplemental videos 5–8).

**Figure 9.**
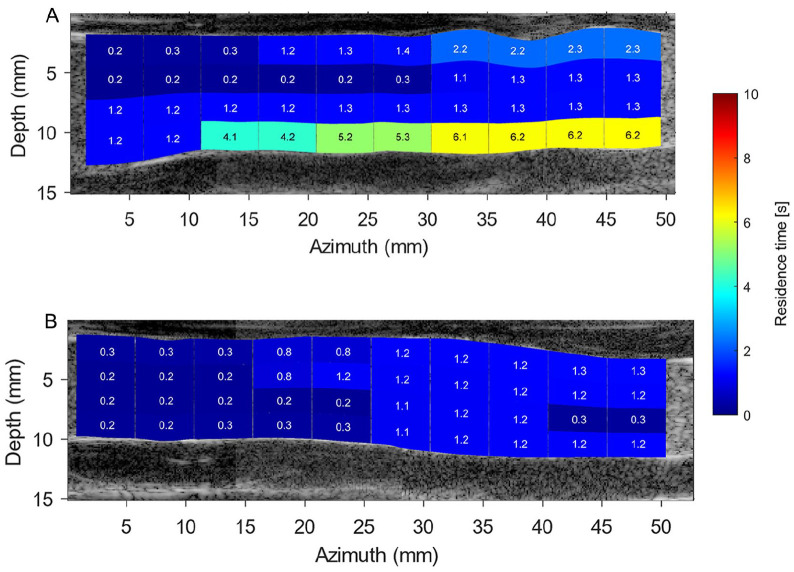
Local residence time (RT) heatmap obtained based on simulated particle trajectories during 10 cardiac cycles in the before endograft placement left (top image) and right (bottom image) iliac arteries. Particles were seeded uniformly in the simulation in each region of interest, and RT was computed as the complete washout duration of all particles from each analysis window. SI 3 contains animations of this simulation (supplemental videos 5 and 6). (A) Before endograft anatomy of left iliac artery. (B) Before endograft anatomy of right iliac artery.

**Figure 10.**
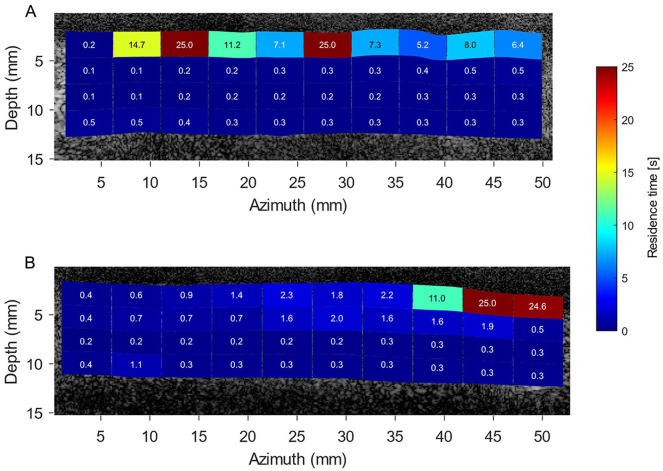
Local residence time (RT) heatmap obtained based on simulated particle trajectories during 25 cardiac cycles in the after endograft placement left (top image) and right (bottom image) iliac arteries. Particles were seeded uniformly in the simulation in each region of interest, and RT was computed as complete washout duration of all particles from each analysis window. SI 3 contains animations of this simulation (supplemental videos 7 and 8). (A) After endograft anatomy of left iliac artery. (B) After endograft anatomy of right iliac artery.

As a showcase, the BE seed particle distributions in the left iliac artery over different time steps are displayed in Figure 8. Residence time heatmaps for BE and AE cases are displayed in Figures 9 and 10, respectively. Particle trajectory simulations performed using PIV velocity fields are presented in supplemental videos 5 to 8 (see SI 3).

Before endograft placement simulations exhibited that left iliac artery had a larger number of regions with higher RT values than presented in right iliac artery. As depicted in Figure 9, a numerical simulation of seed particles exits the right iliac artery within 1.3 seconds, while seed particles stay for a longer time at the posterior wall of the left iliac artery (~6 seconds). The AE RT simulations revealed that seed particles have a preference to stay close to the anterior wall of the left iliac artery, whereas only the anterior wall of the right stented iliac artery upstream to iliac bifurcation demonstrated high RT values (Figure 10). Moreover, although the computation duration of the AE RT simulations is increased until convergence in heatmaps was reached after 25 cardiac cycles, not all seed particles have departed from the stented iliac domains.

## Discussion

This in vitro study indicated the importance of understanding local flow dynamics in relation to thrombotic complications after EVAR. Particularly, the higher VC and longer RT in the left iliac artery with the Anaconda limb, that eventually thrombosed, suggest that this event could have been predicted by having insights on the flow fields in iliac arteries. Based on the data, both anatomical and stent-specific factors likely played a role in this particular case as the disturbances were not caused by the anatomy alone, but the presence of the Anaconda endograft negatively impacting them as well.

The obtained velocity fields at PSV show regions with higher central velocity fields in areas of the iliac anatomy with a decreased cross-sectional area. For instance, downstream of the non-stented left and right iliac arteries (*x*=35–40 mm in Figure 4A, and *x*=40–50 mm in Figure 4B, respectively), local velocities of 85 and 80 cm/s were measured, respectively. The same behavior was observed in AE measurements (Figure 5A-B), confirming that the obtained PIV velocity fields follow continuity, where the velocity is inversely proportional to the vessel cross-sectional area (considering constant mass flow rate).

Due to ballooning of the limb grafts, the iliac arteries have straightened locally, as can be observed in Figure 3. Here, the local wall-to-wall diameter increased at some parts of their anatomy compared to the BE geometry. However, the insertion of the Anaconda limbs led to a local lumen diameter reduction in both limbs, especially in the locations of overlapping rings. Based on the bundle diameter of the Anaconda limbs (~0.4 mm), the lumen limb diameters may be locally reduced by up to 1.6 mm if overlapping rings are present. This may explain the observed increase of the local velocity fields within AE measurements at PSV and ESV time points (Figure 5), respectively, compared to BE measurements (Figure 4).

Vector complexity and RT parameters were computed to provide a quantitative analysis of iliac artery hemodynamics. The BE RT results demonstrate that the seed particles exited the right iliac artery more rapidly (~3-fold faster) resulting in a lower RT than the left iliac. Since, higher RT is considered an unfavorable hemodynamic factor; hence, an a priori prediction BE could be drawn that the left iliac was indeed more prone to LT. It may not be concrete to rely solely to this judgment based on the anatomical similarities of the pre-op and BE iliac arteries; however, these results are proven to be in line with AE findings. The AE RT values are higher in the anterior wall of the left iliac artery including 2 fluid stasis regions. The flow stasis regions are linked to thrombus formation that could eventually cause LT.^18^ On the other hand, the anterior distal wall of the right stented iliac artery also demonstrates large RT values. This could have been a result of the higher density of distal rings since the second right limb graft was placed by compressing the limb graft, positioning the rings closer together, to avoid covering the internal iliac artery. Thus, as a consequence the concertina effect might occur in the right distal ring, causing the observed local fluid stasis (high RT). It should be noted that the distribution of AE RT heatmaps did not follow the same pattern as the BE results, where higher RT values were noticed near the anterior and posterior walls, respectively. The changes in the flow field patterns that occurred by the placement of the endograft can account for this observation.

Vector complexity also provided valuable insights, indicating higher VC values for AE measurements in comparison with BE measurements. Vector complexity patterns are also in line with RT observations, in which more complex flow structures are noticed in the anterior walls of the AE iliac arteries. However, it is not sufficient to use VC norm alone as a LT predictor, due to its dependence on the chosen ROI size. In this study, we used a retrospective approach, in which we set ROI sizes to be the same as the average ring distance. Hence, it is advised to use VC in combination with the RT norm for an improved evaluation or for optimizing the ROI sizes. The VC norm also fails to distinguish between regions characterized by vortical structures and those exhibiting randomly complex velocity fields, for example, due to out-of-plane motion resulting from 2D echoPIV technique. Therefore, future experiments should focus on defining a norm to quantify in-plane vortical structures, and as such allow the quantification of the recirculation zones. Consequently, by discerning these distinct flow features, the reliability of RT outcomes will be improved. Since, regions characterized by a combination of higher vortical structures and longer RT are considered more unfavorable (or pathologically relevant) compared to regions exhibiting high out-of-plane flows and longer RT.^18^ Therefore, by utilizing this approach, proper differentiation of regions with unfavorable hemodynamics can be performed.

Owing to the in vivo feasibility of the echoPIV technique for blood flow quantifications in stented arteries,^25^ future efforts should focus on the evaluation of VC and RT norms in clinical trials with different endograft designs/platforms. Moreover, in vitro follow-up flow studies with (pig) blood mimicking thrombotic events could be useful to further validate the applicability of VC and RT norms for predicting thrombus formation. Also, the influences of implementing different endografts on hemodynamic parameters and potential LT could be explored by using an in vitro flow setup with an averaged AAA phantom, for instance, by calculating the VC and RT norms and also other potential hemodynamic parameters, such as skewedness. Ghanbarzadeh-Dagheyan et al^26^ showed that skewedness parameter could be extracted from 2D echoPIV data to determine the helical flow structures. Therefore, in this proposed study, skewedness could be evaluated in complex AAA anatomical structure, since the helical flow patterns could be induced due to anatomy of an artery or used endografts (or a combination of both). Hence, the underlying concept of the helicity could be investigated and a suitable norm to quantify helicity considering the 2D constrain of current echoPIV technique could be proposed. Furthermore, echoPIV has great potential to be combined with the existing simulation technique, in which finite element analysis and computational fluid dynamics (CFD) simulations were used to provide insights in the hemodynamic assessments after EVAR.^27^ In this case, echoPIV could be used as a tool to provide realistic boundary conditions to improve the CFD simulations, and to validate the simulation results by comparing the echoPIV and CFD flow fields. In this way, a reliable CFD simulation with high temporal and spatial resolution can be obtained, with which complex 3D flow structures, near-wall velocity fields, the influence of fabric infolding in the flow fields, and other details that may not be visible using 2D echoPIV technique can be observed.

After evaluating these hemodynamic norms in future studies, using echoPIV as an affordable and accessible bedside technique in hospitals becomes more promising. Ultrasound particle image velocimetry enables quantification of pre-op flow fields, identifying unfavorable regions and providing valuable insights for surgeons to enhance treatment planning (eg, aid in endograft selection procedure). Ultrasound particle image velocimetry is also applicable for follow-up sessions to evaluate treatment progress and avoid/predict potential complications. Despite the Doppler ultrasound techniques, which require experienced users for accurate 1D flow estimates, echoPIV measurements are angle independent, making it less prone to operator variability. However, to be able to use echoPIV in daily clinical practice, the implementation of the echoPIV technique in commercial ultrasound machines is first needed. Although phase-contrast 4D flow magnetic resonance imaging (MRI)^28^ provides 3D flow field information, it is expensive, time-consuming, averaged over a multiple cardiac cycle, and suffers from metal artifacts, which make it less suitable for post-op. There is also a large trade-off between temporal and spatial resolution in the MRI acquisition compared with the echoPIV technique.

### Limitation

The current single case is exploratory in nature, and thus needs to be confirmed with further studies including other endograft designs, both in vitro and clinically. In this in vitro setup, the feasibility of evaluating the hemodynamic norms with the echoPIV technique was tested in the Anaconda endografts; however, the applicability of this technique is not yet tested for other available commercial endografts. Given the similarities in design with a skeleton consisting of either nitinol or stainless steel and a polyester or polytetrafluoroethylene cover, it is to be expected that the technique can be used in other types of endografts. The flow analysis was limited to a 2D plane in the center of the flow lumen, with potential bias due to out-of-plane motion, leading to the possibility of overlooking flow variations that might exist in other plane slices. The experiments were performed only under physiologic resting conditions, neglecting the influence of different heart rates and variation of physiological pressure fields. Moreover, the prescribed inlet boundary condition assumed an average suprarenal flow profile, which may differ from the actual flow profile of the patient. Although several previous studies used thin-walled aortic aneurysm phantoms from Elastrat for their studies,^29,30^ the used elastic silicone material phantom may have different biomechanical properties than the patient’s aneurysmal wall. For instance, Boersen et al^29^ mentioned that their thin-walled phantoms may be more elastic in comparison with in vivo condition, since aneurysms are usually stiffer because of a larger volume of collagen and less volume of elastin and muscle cells. However, to eliminate the stiffness differences between aortic lumen, thrombus, and calcium, our AAA phantom only represents the flow lumen of the patient (ie, without thrombus and calcification), in which pressure and flow boundary conditions in the inlet (ie, suprarenal artery) and outlet (ie, renal and iliac arteries) were controlled and set to physiological values. The working BMF in the experiment was composed out of a Newtonian fluid solution, disregarding the shear-thinning properties of human blood; however, blood flow in large vessels is considered newtonian.^31^ Flow complexity was determined with VC norm, which is calculated based on velocity vector angles in a defined ROI. Therefore, by altering the ROI size, the VC values may change. For consistency in this study, we divided the flow lumen into several ROIs using the centerline and average Anaconda ring distances. Nevertheless, the optimal selection of ROI, both from a fundamental and clinical standpoint, requires further investigations. Due to uncertainties in PIV measurements near the lumen wall,^32^ velocity vectors in immediate proximity to the wall (ie, first vector after a wall point) were discarded and interpolated by assuming a linear boundary layer profile. As a result, the estimated velocity vector near the wall may differ from the actual value, leading to potential changes in VC and RT values. However, it is expected that the overall trend remains consistent, as the applied assumption aligns with physical reasoning. The inaccuracy of the velocity fields adjacent to the lumen wall is one of the reasons that wall shear stress (WSS) parameters were not calculated in this study. Wall shear stress is calculated based on the velocity gradient, which is more sensitive to noise in the velocity vector estimation than RT and VC norms that are directly dependent on the obtained velocity fields (not their gradients). Furthermore, the desirable WSS values inside endografts and clinical relation between WSS and/or shear rate values with favorable and unfavorable hemodynamic conditions are not yet known, which requires further investigations.^33^

## Conclusion

In conclusion, these in vitro data show that unfavorable hemodynamics are present in the limb that presented with LT, compared to the non-thrombosed side, with higher VC and longer RT. Further studies are needed to unravel the impact of iliac anatomical geometry and endograft design on iliac hemodynamics.

## Supplemental Material

sj-docx-1-jet-10.1177_15266028231219988 – Supplemental material for Ultrasound Particle Image Velocimetry to Investigate Potential Hemodynamic Causes of Limb Thrombosis After Endovascular Aneurysm Repair With the Anaconda Device Supplemental material, sj-docx-1-jet-10.1177_15266028231219988 for Ultrasound Particle Image Velocimetry to Investigate Potential Hemodynamic Causes of Limb Thrombosis After Endovascular Aneurysm Repair With the Anaconda Device by Hadi Mirgolbabaee, Lennart van de Velde, Robert H. Geelkerken, Michel Versluis, Erik Groot Jebbink and Michel M. P. J. Reijnen in Journal of Endovascular Therapy
