# Supplementary material for: Ultrasound Particle Image Velocimetry to Investigate Potential Hemodynamic Causes of Limb Thrombosis After Endovascular Aneurysm Repair With the Anaconda Device
Source: J Endovasc Ther. 2023 Dec 27;32(6):2223–35. doi: 10.1177/15266028231219988 (PMC12598057; doi:10.1177/15266028231219988)
Supplement: sj-docx-1-jet-10.1177_15266028231219988 – Supplemental material for Ultrasound Particle Image Velocimetry to Investigate Potential Hemodynamic Causes of Limb Thrombosis After Endovascular Aneurysm Repair With the Anaconda Device [file sj-docx-1-jet-10.1177_15266028231219988.docx]

**Supplementary Information 1 – Anatomy and endograft details**

In this in-vitro study, a flow phantom was fabricated based on a patient’s infrarenal abdominal aortic aneurysm (AAA) anatomy treated by Anaconda endograft. Since the aim was to quantify the flow field before the development of patient’s left-sided limb thrombosis (LT), the last post-op computed tomography angiography (CTA) scan before LT was used to perform flow lumen segmentation and create the flow phantom. The Anaconda endografts that were deployed in the phantom were identical to the endografts used during that patient's EVAR treatment (see Table SI1.1). Pre-op anatomical features of that patient was extracted based on a modified Eurostar worksheet^1^ (Fig SI1.1) and reported in Table SI1.2.

As depicted in Fig SI1.2, the pre-op and post-op anatomies of iliac arteries were overlapping, representing no significant anatomical differences of iliac arteries after EVAR. Therefore, it was decided to quantify the hemodynamic norms in both stented and unstented iliac arteries.

**Table SI1.1.** Deployed Anaconda endografts during EVAR procedure of the investigated patient with infrarenal AAA.

| **Implant** | **Catalogue Number** |
| --- | --- |
| Main body | ALP30 |
| Left iliac artery | AFL1210x130 |
| Right iliac artery | AFL1210x130 |
| Extension left iliac artery | AL12x80 |
| Extension right iliac artery | AL12x80 |

| 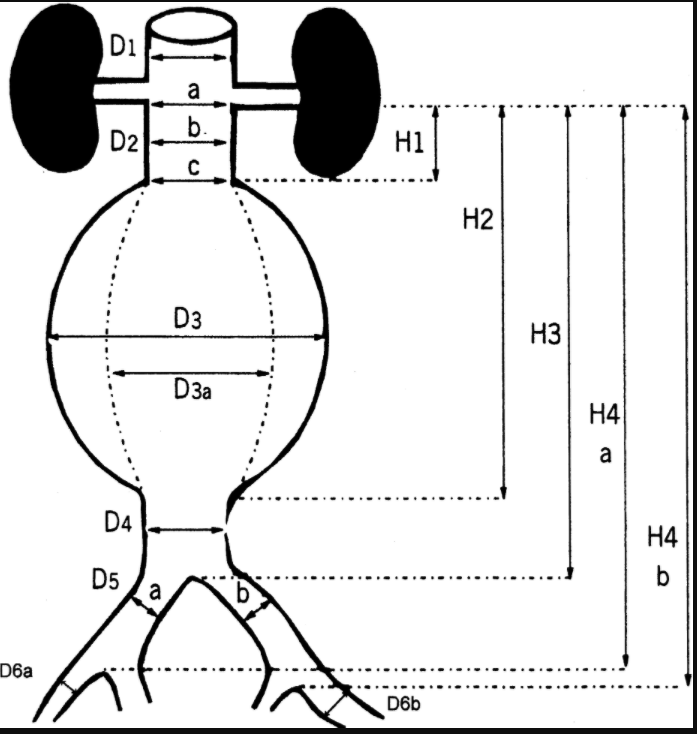 |
| --- |
| ***Fig SI1.1.*** *Infrarenal AAA segments based on Eurostar worksheet^1^.* |

**Table SI1.2.** Extracted pre-op geometrical features of the patient’s AAA. SMA: superior mesenteric artery, RA: renal artery, CIA: common iliac artery, EIA: external iliac artery. Distal and proximal diameters were measured 5mm before and after bifurcations, respectively.

| **D** | **Diameter (mm)** | |
| --- | --- | --- |
| D1 | Aorta diameter between SMA and RA | 24.2 |
| D2a | Aorta diameter just below RA | 28.7 |
| D2b | Between D2a and D2c | 29.1 |
| D2c | Aorta diameter at the end of the infrarenal neck | 30.8 |
| D3 | Max aneurysm wall-wall diameter | 60.4 |
| D3a | Max lumen diameter in AAA sac | 43.9 |
| D4 | Aorta diameter just above the bifurcation | 21.6 |
| D5a | Distal right CIA | 14 |
| D5b | Distal left CIA | 14.1 |
| D6a | Proximal right EIA | 11.2 |
| D6b | Proximal left EAI | 11.4 |
|  | | |
| **H** | **Height (centreline distances in mm)** | |
| H1 | Infrarenal neck | 36.3 |
| H2 | Distance between lowest RA and end of AAA | 98.1 |
| H3 | Distance between lowest RA and aortic bifurcation | 116.3 |
| H4a | Distance lowest RA and right CIA bifurcation | 197.5 |
| H4b | Distance lowest RA and left CIA bifurcation | 206.4 |

| 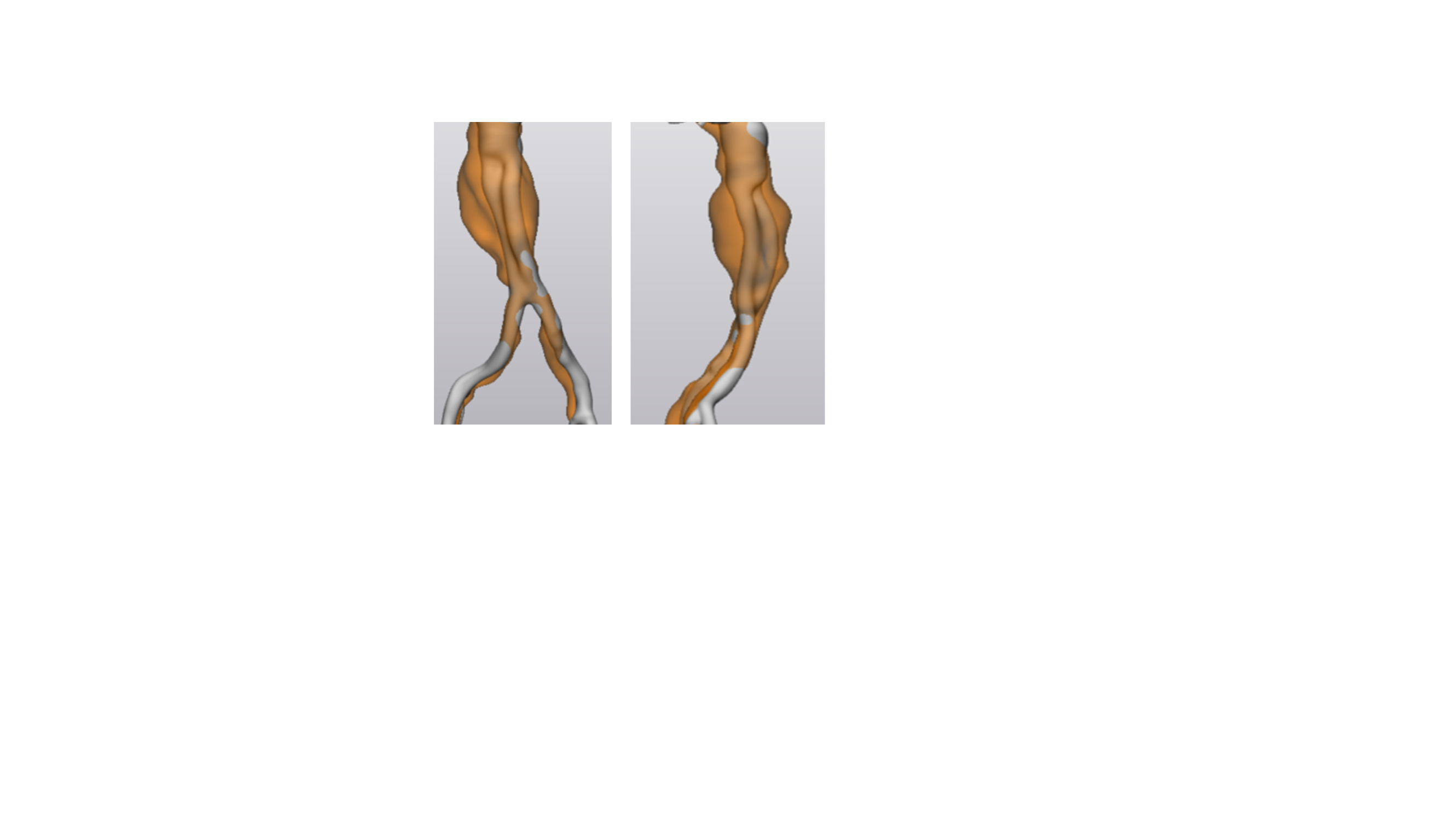 |
| --- |
| ***Fig SI1.2.*** *Pre-op and post-op anatomies of the iliac arteries, which were overlapped on each other. The orange and white colors represent the pre-op and post-op anatomies, respectively.* |

**Supplementary Information 2 – EchoPIV, filtering, analysis details**

***Ultrasound particle image velocimetry (echoPIV)***

L12-3V probe was programmed to acquire high frame rate (HFR) contrast-enhanced plane wave ultrasound (CEUS) images using two apertures to capture the maximum lumen length withing each measurement (~38mm). The unstented (referred as before endograft placement (BE)) and stented (referred as before endograft placement (AE)) echoPIV measurements were performed at 2000 and 6250 frames per second (fps), respectively. Although the flow setup and boundary conditions were the same for BE and AE measurements, higher fps was proven to be necessary for singular value decomposition clutter (SVD) filtering to eliminate double-layer stent signal (refer to filtering subsection).

***Filtering***

Singular value decomposition clutter (SVD) filtering^2^ was used to preprocess the radiofrequency (RF) data. First, the Casorati matrix ($n\times m\times np$) was formed, where “n” is the total number of samples received from each channel, and “m” is the total number of acquired frames, and “np” represents the total number of acquisition apertures. After decomposing the Casorati matrix, manual cut off ranges were selected based on the principal component strength figure to eliminate the signals from the phantom walls and stents. Thereafter, image reconstruction was done using the filtered RF data, which were used as an input for our PIV analysis workflow (refer to Analysis subsection). In general, the effectiveness of SVD in distinguishing image features is enhanced when a larger dataset is used. Since more random reflections were noticed in the raw ultrasound images during AE measurements, due to double-layer limb grafts, we increased the fps to create a larger dataset of US images.

***Analysis***

Vector complexity (VC) quantifies the complexity of flow field inside a chosen region based on the spread of the velocity field directions. Pedersen et al. presented a detail explanation of VC’s mathematical implementations^3^. Due to the averaging nature of the VC norm, the main challenge is to establish a proper way to divide the flow lumen. In this study, the flow lumen was equally divided in the horizontal and vertical direction to form a 4*10 region of interest (ROI), Fig 3 in the manuscript, based on the flow lumen centerline and average Anaconda limb graft ring distances in the AE condition. Therefore, a retrospective approach was chosen to define the horizontal division of the flow lumen.

Rayz et al. presented a detail explanation of the residence time (RT) simulations^4^. In this study, RT simulations were performed by initializing the flow lumen with mass-less particles, which were positioned on the obtained PIV grid points across the flow lumen. Thereafter, RT simulations were performed based on the temporal resolution and scattered interpolation of the velocity fields obtained from PIV results. During each time point, particles position was recorded. Finally, when all particles left an ROI, an RT value was reported. It should be noted that the flow lumens were divided with the same ROIs as it was done for VC measurements.

**Supplementary Information 3 – Videos**

echoPIV and RT simulations videos are only visible in the online version:

- Video 1: BE velocity fields obtained from left iliac artery.
- Video 2: BE velocity fields obtained from right iliac artery.
- Video 3: AE velocity fields obtained from left iliac artery.
- Video 4: AE velocity fields obtained from right iliac artery.
- Video 5: RT simulation based on BE velocity fields obtained from left iliac artery.
- Video 6: RT simulation based on BE velocity fields obtained from right iliac artery.
- Video 7: RT simulation based on AE velocity fields obtained from left iliac artery.
- Video 8: RT simulation based on AE velocity fields obtained from right iliac artery.

**References**

1. Rödel S, Geelkerken RH, Van Herwaarden JA, et al. Consistency in endovascular aneurysm repair suitability assessment requires group decision audit. Journal of Vascular Surgery. 2006;43(4):671-676. doi:10.1016/j.jvs.2005.11.055
2. Demene C, Deffieux T, Pernot M, et al. Spatiotemporal clutter filtering of ultrafast ultrasound data highly increases Doppler and fUltrasound sensitivity. *IEEE Transactions on Medical Imaging*. 2015;34(11):2271-2285. doi:10.1109/tmi.2015.2428634
3. Pedersen MM, Pihl MJ, Haugaard P, et al. Novel flow quantification of the carotid bulb and the common carotid artery with vector flow ultrasound. *Ultrasound in Medicine & Biology*. 2014;40(11):2700-2706. doi:10.1016/j.ultrasmedbio.2014.06.001
4. Rayz VL, Boussel L, Ge L, et al. Flow residence time and regions of intraluminal thrombus deposition in intracranial aneurysms. *Annals of Biomedical Engineering*. 2010;38(10):3058-3069. doi:10.1007/s10439-010-0065-8
